# Editorial: Human resource management in the COVID-19 era: new insights and management opportunities

**DOI:** 10.3389/fpsyg.2023.1161524

**Published:** 2023-04-17

**Authors:** Diletta Gazzaroli, Caterina Gozzoli, Natalia Garcia-Carbonell, Gonzalo Sanchez-Gardey

**Affiliations:** ^1^Department of Psychology, Catholic University of the Sacred Heart, Milan, Italy; ^2^Organización de Empresas, University of Cádiz, Cádiz, Spain

**Keywords:** human resources management (HRM), COVID-19, new HRM perspective, new HRM practices, new HRM challenges

Today's human resource management professionals need to deploy a complex set of competencies to deal with different issues that are threatening organizations' performance and even their survival.

The COVID-19 pandemic set off a situation that involves a rupture with the past, which has made HRM's fragility and challenges—some of which already existed—emerge in an explosive and faster way. The current scenario can be defined in terms of ambiguity, uncertainty, precariousness, instability, and new possibilities as it never has been before.

Changes in traditional work processes and conditions have shaped an environment in which human resource management (HRM) is called on to face issues that range from changes in the way people work and interact in the workplace (rapid digitization, remote and virtual environments, groups, and team working) to the deep representations and meanings related to work, to foster productivity, innovation, and wellbeing.

HRM has recently been required to face important challenges because of the increasing complexity of organizations and the multidimensional nature of work. Despite all the advances that have been made in HRM studies, the social and economic effects of the COVID-19 crisis put researchers and practitioners in a position in which they face a lack of framework, procedures, and tools to guide and support professionals' adjustment to all the drastic changes that happened in the work and social environment.

A broader and more interdisciplinary view is required to understand and take on HRM's changes and challenges and to find new and integrated ways through which HRM can safeguard wellbeing, innovation, and productivity in organizations.

This Research Topic aimed to provide a constructive and reflexive debate on HRM, bringing together current advances on the topic from a multidisciplinary perspective. We looked for studies both theoretical and empirical (quantitative, qualitative, or mixed-method approaches), from different domains such as psychology, management, sociology, and related fields. This Editorial aims to provide an overview of the interesting contributions that have been published on the merits of this Research Topic. Thus, the complexity of the topic certainly has encouraged a rich and substantial scientific debate, and thanks to the authors, some enlightening areas for reflection can be outlined, including the importance of complex and multifocal HRM approaches and directing actions and choices toward the employees.

As three of the contributions to this Research Topic point out (Cao et al.; Chen; Fregnan et al.), competencies are a hot topic, not only in terms of the need for more complex conceptualization but also in terms of rethinking and reconfiguring the prospects for their development and capitalization.

If we assume competencies as comprising a combination of knowledge, skills, and experiences that are useful for people in a broader perspective—including conscious participation in every aspect of daily life experience, as well as the creation of a new future dimension with intention and full awareness, it is clear why there is a growing trend toward recognizing employees as a lever for organizational innovation. This implies a new, different perspective in terms of mutual expectations between organizations and employees.

However, the awareness of the value of employees comes with the question of how to capitalize on the skills and knowledge that professionals develop considering the ever-increasing need to reexamine and revive the continuity of the individual–organization relationship.

In regard to this new relationship between employees and organization, five of the contributions published in this Research Topic provide insights that go in the direction of giving attention to the individual, giving voice to every role, and valuing the specificities of each professional in the company (Liu, Liu et al.). Thus, it becomes crucial to identify factors that can support workers' perceptions of self-efficacy and safety (Wang et al.). These include assuming a 360° view of the employee that takes into consideration the person-environment fit (i.e., the development of the person's potential and more relational dimensions than performance alone), the importance of clear boundaries and giving voice/space to workers and their needs (building dialogue and theme of mutual accountability), and the importance of middle management roles, especially from a relationship perspective (Jiang et al.; Mellner et al.; Wang).

The outlined scenario undoubtedly points to the need for a more central and strategic function of HRM roles. The contributions of Liu, Zhang et al., Shan et al., and Zhao et al., emphasize that this complex approach to resource management should combine multiple looks. An approach geared toward promoting worker expression, providing support, developing meaningful relationships, and maintaining a culture of error that does not condemn is a decisive factor in supporting individual creativity.

Research tells us that, for people, the organizational experience can no longer be traced back to (and be limited to) being “just” executive assets. Employees are looking for (and asking for) meaningfulness that connects purpose, process, and performance. People bring the need to perceive themselves as protagonists in their daily professional lives and to recover a sense of fulfillment in what they do. This is also related to the progressive loss of a sense of belonging to the organization, resulting in an impoverishment of the company's assets.

We believe that for HR practitioners, the question that should permeate functions and planning is: “What is the new meaning of work?” Therefore, in terms of application, it is essential not to take old paradigms for granted but renew the pact with workers and understand what they are looking for beyond economic benefits.

One last piece of food for thought is the thematization of group dimensions and relational connections among employees. In most cases, the desire for enhancement and contact is declined regarding the individual and the individual's relationship with top management. These data tell us, implicitly, to remember that there is a substantial difference between valuing *individuality* and valuing *individualism* and we need to find a balance between the individual and the more systemic dimension and nurture both.

## Author contributions

All authors listed have made a substantial, direct, and intellectual contribution to the work and approved it for publication.

